# Hybrid dark-field and attenuation contrast retrieval for laboratory-based X-ray tomography

**DOI:** 10.1364/OPTICA.525760

**Published:** 2024-11-25

**Authors:** Adam Doherty, Ian Buchanan, Oriol Roche i Morgó, Alberto Astolfo, Savvas Savvidis, Mattia F. M. Gerli, Antonio Citro, Alessandro Olivo, Marco Endrizzi

**Affiliations:** 1 Department of Medical Physics and Biomedical Engineering, University College London, London, WC1E 6BT, UK; 2 X-ray Microscopy and Tomography Lab, The Francis Crick Institute, 1 Midland Road, London, NW1 1AT, UK; 3 UCL Division of Surgery and Interventional Science, Royal Free Hospital, NW3 2PF, London, UK; 4 Stem Cell and Regenerative Medicine Section, Great Ormond Street Institute of Child Health, University College London, London, WC1N 1EH, UK; 5 San Raffaele Diabetes Research Institute, IRCCS San Raffaele Scientific Institute, Milan, Italy

## Abstract

X-ray dark-field imaging highlights sample structures through contrast generated by sub-resolution features within the inspected volume. Quantifying dark-field signals generally involves multiple exposures for phase retrieval, separating contributions from scattering, refraction, and attenuation. Here, we introduce an approach for non-interferometric X-ray dark-field imaging that presents a single-parameter representation of the sample. This fuses attenuation and dark-field signals, enabling the reconstruction of a unified three-dimensional volume. Notably, our method can obtain dark-field contrast from a single exposure and employs conventional back projection algorithms for reconstruction. Our approach is based on the assumption of a macroscopically homogeneous material, which we validate through experiments on phantoms and on biological tissue samples. The methodology is implemented on a laboratory-based, rotating anode X-ray tube system without the need for coherent radiation or a high-resolution detector. Utilizing this system with streamlined data acquisition enables expedited scanning while maximizing dose efficiency. These attributes are crucial in time- and dose-sensitive medical imaging applications and unlock the ability of dark-field contrast with high-throughput lab-based tomography. We believe that the proposed approach can be extended across X-ray dark-field imaging implementations beyond tomography, spanning fast radiography, directional dark-field imaging, and compatibility with pulsed X-ray sources.

## 1. INTRODUCTION

In X-ray dark-field and phase-contrast imaging, contrast is generated by the phase changes imparted to the radiation as it traverses the sample. Sensitivity to these contrast channels requires the use of specialized setups, often implemented with synchrotron radiation that offers high flux and coherence, but also adapted to laboratory-scale equipment. We focus here on edge illumination [1], for its applicability with rotating anode X-ray sources [2], robustness [3], and negligible coherence conditions (both spatial and temporal) [4] and because it is an approach that allows for uniform sampling of the illumination across a cm-sized field of view [5], which is important for single-shot imaging.

In phase-sensitive X-ray setups, the intensity reaching the detector is modulated by both attenuation and phase effects and separating these signals into attenuation, (differential) phase, and dark-field images typically requires taking multiple exposures under different conditions. Such examples include changing the propagation distance [6], rocking of a crystal analyzer [7], lateral movement of phase gratings [8–10], lateral movements of diffusers [11–14], or lateral movements of absorbing masks [15]. Alternatively, this can be done with a single exposure by finely sampling the wavefront but comes at a cost of reducing the system resolution below that of the detector pixel size, which has been shown with radiation structured by interference gratings [16], diffusers [17], structured grids [18,19], and absorbing masks [20]. However, such techniques often make use of multiple exposures to compensate for the resolution loss from a single-shot approach [21–23].

Single-shot approaches to phase-contrast imaging that do not rely on high-resolution detectors often rely on the assumption of a homogeneous object approximation, which links the real and imaginary parts of the refractive index of the material under investigation [24]. This has recently been expanded in the case of single-shot dark-field retrieval using synchrotron radiation [25,26]. We use a similar approach, but make use of a macroscopic-homogeneous object approximation to instead link the attenuation and dark-field signals. This is demonstrated with a lab-based system, and we show the benefits on the data-intensive application of tomography acquisition and reconstruction.

In this work we introduce a novel retrieval method to extract dark-field contrast with a single measurement. The laboratory-based system is first described in Section 1.A. Then we show our model that allows retrieval of the dark-field contrast in a single exposure in the case of a pure phase object in Section 2. We then extend this to moderately attenuating but macroscopically homogeneous samples for a hybrid attenuation and dark-field contrast for tomography in Section 2.A. Finally, we discuss the validity of this approach and show example tomographic reconstructions with biological samples in Section 3.

### A. Edge-Illumination Setup

**Fig. 1.**
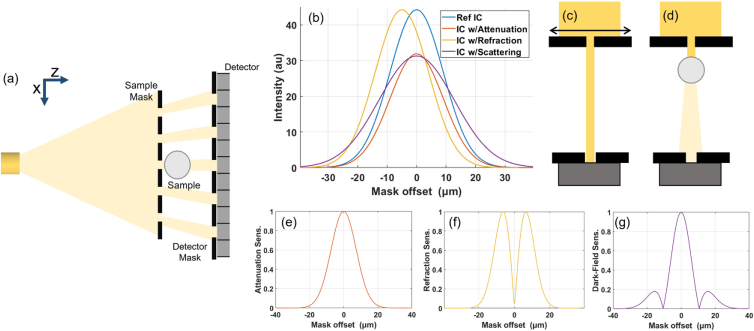
(a) Edge illumination system schematic with radiation from an X-ray tube source modulated by the sample mask before reaching the sample, and then travelling towards the detector mask and detector. (b) Typical illumination curves (IC), i.e., the intensity acquired by one pixel when the sample mask is translated along the *x*-axis through one period [see arrow in (c)], and the effect on an illumination curve for purely attenuating, refracting, and scattering samples. (c), (d) Single beamlets are shown for a pictorial description of those effects, for the mask position corresponding to the peak in the illumination curve. A drop of intensity is expected at this position from both attenuation as well as scattering effects, associated with the dark-field signal. Sensitivity to the effects of (e) attenuation, (f) refraction, and (g) dark-field scattering as a function of the illumination curve position. These are calculated from the normalized magnitude of the intensity change from a small signal from each channel.

A schematic of an edge illumination system is shown in Fig. 1(a). The system uses an absorbing mask that has a one-dimensional periodic structure that modulates the beam into a series of independent beamlets. These beamlets are then absorbed, refracted, and scattered by the sample. A second mask is placed just before the detector to analyze the profiles of these beamlets by creating insensitive regions on the detector pixel, and thus scattered radiation can be selectively measured by moving the sample mask to misalign the beamlets with the detector mask aperture. The one-dimensional shape means that phase sensitivity is along the direction between beamlets, and each beamlet is associated with one detector pixel.

The experiments in this work were performed with the prototype system developed at UCL. This setup comprises an X-ray source, detector, and two periodic masks. The X-ray source is a Rigaku MicroMax-007 HF (Rigaku, Japan) with a molybdenum rotating anode and an effective focal spot size of approximately 70 µm, which was operated at 40 kVp and 30 mA for all results presented here. No additional filtration to the inherent 200 µm Be window was used. The detector was a CMOS-based flat-panel C9732DK-11 (Hamamatsu, Japan) with $50\times 50\;{\text{µ}m}^{2}$ pixels and was placed at 85 cm from the source. The sample mask was placed at 68.5 cm from the source. Masks were made of Au on a graphite substrate and were manufactured, to the authors’ design, by Creatv MicroTech (Potomac, MD). The sample mask had an aperture size of 10 µm and a period of 79 µm, while the detector mask had an aperture size of 17 µm and a period of 98 µm. The periods match each other after taking into account the geometric magnification. The detector mask was placed just in front of the detector with the mask period matching twice the detector pixel period, after projection onto the detector plane. Both masks were in total 400 µm thick, consisting of a graphite substrate, and the absorbing sections of the masks made of 120 µm of Au. The sample was placed just behind the sample mask, leading to a geometric magnification of approximately 1.2. A full characterization of the system can be found in Havariyoun *et al.* [27].

Imaging with the edge illumination system is through the acquisition and processing of each pixel’s illumination curve. This is the intensity measured by one pixel as the sample mask and sample are translated in one period, creating an offset between the sample mask and the detector mask and detector. A series of exposures is taken during this procedure at different positions between the sample mask and the detector mask along the *x*-axis—with the resulting measurements for each pixel forming the illumination curve. A model illumination curve is shown in Fig. 1(b) with the effects of attenuation, refraction, and scattering demonstrated if a sample was placed in the beam. A Gaussian function is a well-established approximation for the illumination curve [15]. However, a Gaussian poorly captures the tails of the illumination curves, with an “offset” occasionally used to improve the retrieval [28,29]. The exact origin of the offset is not well understood and is higher than the expected transmission through the masks of roughly 1% at our energy and mask dimensions. The dark-field image is retrieved through fitting Gaussian functions to the data acquired for each illumination curve [30], with the change in the area under the illumination curve corresponding to attenuation, the shift in center position to refraction or differential phase, and the increase in width to scattering or dark-field contrast. In the case of tomography [31], the procedure needs to be repeated for each viewing angle, and thus the amount of images required is the number of sampling points along the illumination curve times the number of viewing angles, $N_{IC}\times N_{proj}$ .

In an edge illumination system, spatial resolution can be improved through a procedure known as dithering, where the sample is translated in small steps along the *x*-axis, subsequently exposing parts of the sample that have not been illuminated. In the case of full illumination curve sampling, this further increases the acquisition dimensionality to three and the number of exposures to $N_{IC}\times N_{dith}\times N_{proj}$ . It should be noted that tomography is possible without dithering with a single exposure per projection angle [32]. Here, the spatial resolution will be ultimately limited by the pixel size, as with conventional X-ray imaging systems [33]. Furthermore, techniques are under development for retaining this spatial resolution improvement without fully dithering the sample for reducing the number of necessary exposures [34].

The model and methodology presented here allow retrieval of dark-field contrast in a single shot with a stationary sample mask (i.e., no illumination curve scanning, $N_{IC}=1$ ), reducing the acquisition dimensionality by one for all dark-field acquisitions. The proposed method additionally does not require curve fitting and hence it also substantially speeds up an otherwise computationally intensive data processing.

## 2. SINGLE-SHOT DARK-FIELD MODEL

The basis for single-shot dark-field imaging with edge illumination is a convolution model of the illumination curve. The illumination curve without the sample is denoted as the reference $I_{r}(\bar{x})$ :

(1) $$I_{r}(\bar{x})=\frac{A}{\sqrt{2\pi \sigma_{r}^{2}}}\exp\;\left[-\frac{{\left(\bar{x}-{\bar{x}}_{0}\right)}^{2}}{2\sigma_{r}^{2}}\right],$$

where $\bar{x}$ indicates the displacement of the sample mask along the *x*-axis, *A* is a constant determining the amplitude of the curve, $\sigma_{r}^{2}$ is the variance (width) of the Gaussian curve, and ${\bar{x}}_{0}$ is the position of alignment between the sample and the detector apertures These parameters all define the shape of the illumination curve without the sample, and are dependent on source power, mask aperture sizes, system geometry, and detector efficiency. Note that these parameters $\left[A,{\bar{x}}_{0},\sigma_{r}^{2}\right]$ are all pixel-wise and vary between illumination curves across the detector. Variations in ${\bar{x}}_{0}$ can originate from mask imperfections or misalignment. Single-shot dark-field retrieval requires imaging at the same point on each illumination curve across the detector, meaning variations in ${\bar{x}}_{0}$ should be minimized but these can be reduced to well below a micron across the whole field of view [5,35]. We will assume ${\bar{x}}_{0}$ to be constant for all pixels. Variations in $\sigma_{r}^{2}$ are less critical and can be accounted for by pixelwise correction, but we can assume this parameter to be constant without a significant performance loss.

When a sample is in the beam, the illumination curve is denoted as the sample illumination curve $I_{s}(\bar{x})$ and includes three additional terms:

(2) $$I_{s}(\bar{x})=\frac{tA}{\sqrt{2\pi (\sigma_{r}^{2}+\sigma_{o}^{2})}}\exp\;\left[-\frac{{\left(\bar{x}-{\bar{x}}_{0}-\Delta {\bar{x}}_{ref}\right)}^{2}}{2(\sigma_{r}^{2}+\sigma_{o}^{2})}\right],$$

with *t* quantifying the change in area from attenuation, $\Delta {\bar{x}}_{ref}$ quantifying the lateral shift from refraction, and $\sigma_{o}^{2}$ quantifying the broadening in angular distribution from scattering (dark-field). Calculating these three parameters on a pixel-by-pixel basis yields attenuation, refraction, and dark-field contrast images.

In the convolution model, the dark-field signal, quantified by $\sigma_{o}^{2}$ , is the width of the scattering function associated with each part of the sampled object. As such, dark field is measured in units of length squared and is then converted to squared angle distribution by dividing by the squared propagation distance. This approach for the measurement of dark field signals then is independent of system geometry [36]. The convolution model is also used in grating interferometry [10,37–39], speckle-based imaging [12,40], and single-grid imaging [22,41,42]. However, such systems generally measure a visibility reduction associated with dark-field blurring and can then optionally convert this into a scattering angle. For a system based on interferometric methods, such conversions will only yield a stable value when the scattering feature size is smaller than the system’s autocorrelation length [43]. In edge-illumination, where sampling beamlets do not interact with each other, the autocorrelation length is typically less than 1 µm and as such, the retrieved scattering angle tends to be consistent despite changes in mask period [36]. To our knowledge, there is only one publication that experimentally compares edge-illumination dark-field measurements to other phase sensitive systems, this time to dark field with speckle-based imaging, which found a poor qualitative match between the two systems [44]. However a full comparison is difficult due to the broad range of different algorithms published to retrieve dark field with interference-based dark-field imaging.

The effects of the attenuation and refraction signals must be isolated or excluded for retrieving $\sigma_{o}^{2}$ from a single measurement. The sensitivity to each contrast channel is a function of the mask offset and hence can be optimized accordingly based on where on the illumination curve this measurement it taken. This is presented through a simple model in Figs. 1(e)–1(g), where the absolute change in intensity is plotted across the illumination curve for a small signal from each of the three contrast channels, calculated as $\frac{∥I_{r}(\bar{x})-I_{s}(\bar{x})∥}{\sqrt{I_{r}(\bar{x})}}$ . Strictly speaking these sensitivity curves depend on the absolute signal in each contrast channel; however the sensitivity plots we report are typical curves given the signal level experienced in many imaging cases. Sensitivity to refraction is linked to the gradient of the illumination curve [45], and hence it is minimized at the peak and tails, which is also where sensitivity to the dark-field broadening is highest. Sensitivity to the attenuation signal is highest at the peak. For single-shot dark-field imaging, we chose to expose at the peak of the illumination curves [see Fig. 1(d)]. At this position, the intensity changes when the illumination curve arising from a lateral shift is minimized, whereas the change due to broadening is maximized. We also note that this illumination is the most dose-efficient with little X-ray intensity lost in the detector mask.

At the peak of the illumination curve, i.e., when $\bar{x}={\bar{x}}_{0}$ , the measured signals for the reference and sample illumination curves are as follows:

(3) $$I_{r}(\bar{x}={\bar{x}}_{0})=\frac{A}{\sqrt{2\pi \sigma_{r}^{2}}},$$

(4) $$I_{s}(\bar{x}={\bar{x}}_{0})=\frac{tA}{\sqrt{2\pi (\sigma_{r}^{2}+\sigma_{o}^{2})}}\exp\;\left[-\frac{\Delta {\bar{x}}_{ref}^{2}}{2(\sigma_{r}^{2}+\sigma_{o}^{2})}\right].$$

The assumption for single-shot dark-field imaging is that the shift in the center position is small compared to the width of the curve, i.e., $\Delta {\bar{x}}_{ref}^{2}\ll \sigma_{r}^{2}$ . This comes about from the peak of the illumination curve being flat, meaning a small lateral shift will not lead to a large change in intensity. As such, refraction effects can be neglected and sensitivity to scattering is retained, reducing the number of unknowns to two (*t* and $\sigma_{o}^{2}$ ).

The validity of this assumption depends on the system and sample being scanned. For a system with relatively wide apertures, the illumination curves will be broad (large $\sigma_{r}^{2}$ ) and hence larger refraction angles could be tolerated. In terms of the sample, refraction occurs at material boundaries, whereas attenuation and scattering typically happen in the bulk of the sample, although some scattering is seen at material boundaries [25]. For the system being employed here, this assumption is valid away from material boundaries, where some intensity change is likely to come from refraction.

The above conditions allow for a simplification in Eq. (4) where $\exp[-\frac{\Delta {\bar{x}}_{ref}^{2}}{2(\sigma_{r}^{2}+\sigma_{o}^{2})}]\approx 1$ . The following step involves taking the ratio between Eqs. (3) and (4) and squaring the result to obtain

(5) $$\frac{I_{r}^{2}(\bar{x}={\bar{x}}_{0})}{I_{s}^{2}(\bar{x}={\bar{x}}_{0})}=\frac{\sigma_{r}^{2}+\sigma_{o}^{2}}{t^{2}\sigma_{r}^{2}}.$$

Rearranging this to solve the dark-field signal gives the equation for dark-field retrieval:

(6) $$\sigma_{o}^{2}=\sigma_{r}^{2}\left[t^{2}\frac{I_{r}^{2}(\bar{x}={\bar{x}}_{0})}{I_{s}^{2}(\bar{x}={\bar{x}}_{0})}-1\right],$$

and defining $\Omega =\frac{I_{r}^{2}(\bar{x}={\bar{x}}_{0})}{I_{s}^{2}(\bar{x}={\bar{x}}_{0})}$ this can be written in more compact form as

(7) $$\sigma_{o}^{2}=\sigma_{r}^{2}(t^{2}\Omega -1),$$

with the dark-field signal expressed as a function of the width of the reference illumination curves, $\sigma_{r}^{2}$ , the transmission signal, *t*, and the change in peak illumination curve measurement, quantified as Ω .

In the case of a pure-phase object where t=1 , then Eq. (7) allows the retrieval of the dark-field signal with a single exposure. As has been shown previously, the dark-field signal recovered with the edge-illumination system is compatible with tomography [31,46], and hence this approach allows retrieval of dark-field contrast with weakly attenuating samples with a single shot.

To demonstrate the compatibility with tomography we present the signal from a foamed polystyrene wedge that only produced around 1.5% attenuation at its thick edge. The dimensions of the wedge are roughly 16×10mm along the x and *z* axes, respectively. At the energy employed here, the refractive index terms are $\delta =1.37\times 10^{-8}$ and $\beta =5.35\times 10^{-12}$ [47]. This was scanned with 16 dithering steps and 1.2 s exposure per frame, with nine illumination curve points taken centered around the peak for conventional dark-field retrieval, with the peak measurement used for single-shot retrieval.

**Fig. 2.**
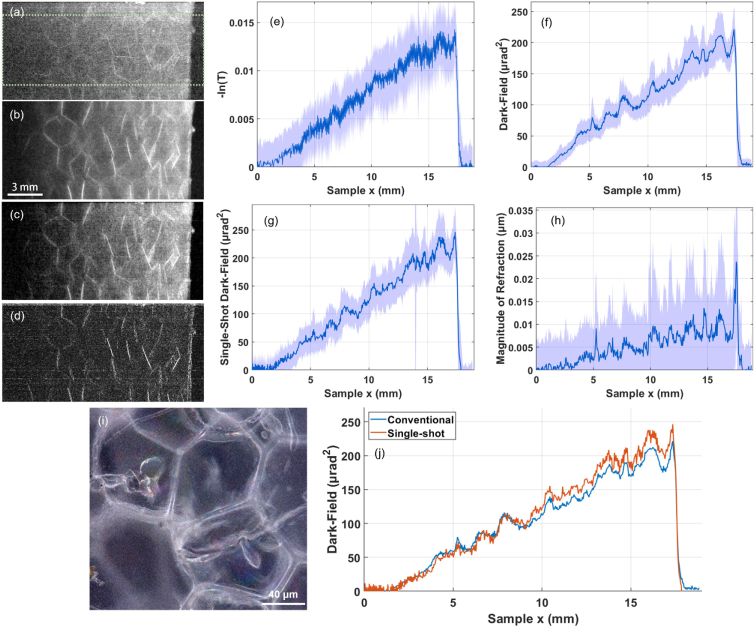
Images and profiles of polystyrene wedge phantom with (a)+(e) attenuation contrast, (b)+(f) dark-field contrast, (c)+(g) single-shot contrast using Eq. (7), assuming t=1 , and (d)+(h) the absolute value of the refraction signal, $|\Delta {\bar{x}}_{ref}|$ . Profiles are taken by averaging all rows within the green ROI, with error bars taken as the standard deviation in the *y*-axis of the image, and graph horizontal axes correspond the to *x*-coordinates of the image. The sub-resolution features are resolved in (i) using a light microscope. Conventional and single-shot dark-field contrast are shown together in (j). The profiles match exactly at the thinnest edge due to the lack of attenuation, and start to deviate as the sample shows higher attenuation as the wedge thickness increases, breaking the assumption of a pure-phase object.

Images and profiles for this wedge in the dark-field channel retrieved using conventional and single-shot retrievals are presented in Fig. 2. These both show good linearity and the shape is consistent between the two profiles across the full wedge, but a small discrepancy between them [Fig. 2(j)] is present. This is partially from the assumption of a pure phase object not being true ( t∼0.99 at the thickest edge), but is also potentially arising from $\sigma_{r}^{2}$ varying across the profile, mask misalignment across the field of view, and errors in the conventional multi-point dark-field retrieval [48]. Overall there is uncertainty when obtaining quantitative dark-field retrieval with any approach, but in this weak-attenuation regime the single-shot retrieval results in a profile that roughly matches that from conventional dark-field retrieval.

### A. Extension to Tomography with Attenuating Samples

**Fig. 3.**
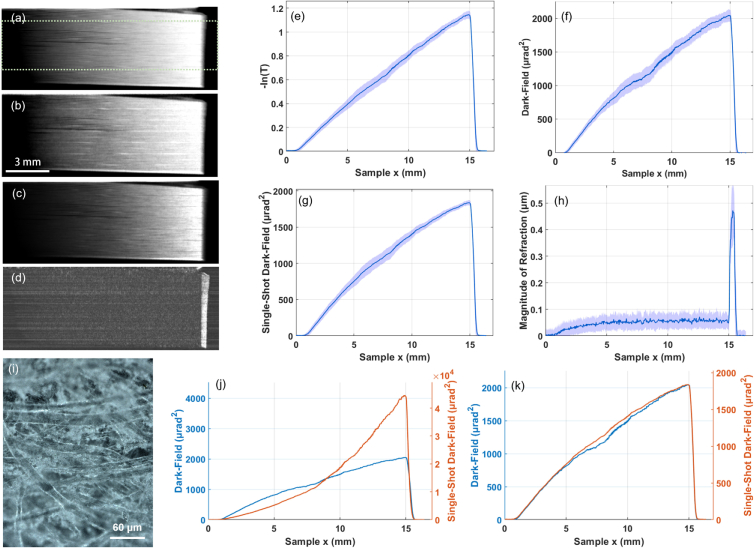
Images and profiles of paper wedge phantom with (a)+(e) attenuation contrast, (b)+(f) dark-field contrast, (c)+(g) single-shot hybrid contrast using Eq. (14) with $\gamma =551\;{mrad}^{-2}$ , and (d)+(h) the absolute value of the refraction signal, $|\Delta {\bar{x}}_{ref}|$ . Profiles are taken by averaging all rows within the green ROI, with error bars taken as the standard deviation in the *y*-axis of the image, and plot horizontal axes correspond to the *x*-coordinates of the image. The sub-resolution features of the paper fibers are resolved in (i) using a light microscope. Additionally in (j) we show the signal retrieved using Eq. (7) with t=1 alongside the conventional dark-field signal, where a large discrepancy is found and the signal is overestimated due to the non-negligible contribution from attenuation that is not accounted for. We show the same in (k) for the linearized single-shot hybrid contrast, where a linear signal that is much closer to the conventional dark-field signal is retrieved.

Solving Eq. (7) to obtain a dark-field image with a single exposure of the sample still requires knowledge of the transmission, *t*. An initial approach is the assumption of a phase object, where transmission can be assumed to be unity. For samples showing non-negligible attenuation, simply substituting t=1 in Eq. (7) results in a signal that is unsuitable for tomography because it cannot be expressed as an integral as the X-ray path through the sample [see Fig. 3(j)]. Reconstructing this signal would lead to cupping artifacts, where the center of absorbing objects will be brighter or darker than expected, similar to those seen with beam hardening. Non-linear projection intensity is common in polychromatic X-ray tomography and reconstruction algorithms exist to account for this [49,50]; however, our dependence on thickness is superlinear rather than the sublinear signal present with beam hardening. Furthermore, these methods tend to be model specific, and hence a dedicated model based on the intensity measured with dark-field sensitive setups is required.

We propose an approach based on a relation between the attenuation and dark-field signals to linearize the single-shot contrast and reduce our model to a single unknown. We can constrain the solution of Eq. (7) by imposing a macroscopically homogeneous material approximation where the ratio between the attenuation and scattering signal is constant across the sample. We start by recognizing that both the attenuation signal and dark-field signal can be expressed as the linear integral along the X-ray propagation axis:

(8) −ln[t(x,y)]=∫μ(x,y,z)dz,

(9) $$\sigma_{o}^{2}(x,y)=\int \epsilon (x,y,z)\;dz,$$

where [x,y,z] represent the system coordinates, μ is the linear attenuation coefficient, and ϵ is the linear scattering coefficient that can be reconstructed in edge-illumination dark-field tomography [31].

For a material with a constant relation between attenuation and dark-field scattering, we can define a new parameter:

(10) $$\gamma (x,y,z)=\frac{\mu (x,y,z)}{\epsilon (x,y,z)},$$

which will be constant across the sample as with the definition of a macroscopically homogeneous sample (i.e., γ=γ(x,y,z) ). We now take the ratio between the retrieved attenuation and dark-field projections as

(11) $$\frac{-ln[t(x,y])}{\sigma_{o}^{2}(x,y)}=\frac{\int \mu (x,y,z)dz}{\int \epsilon (x,y,z)dz}.$$

Inserting Eq. (10) into the above and noting that *γ* being a constant allows the removal of the integral and position dependencies, we end up with the following definition of *γ* in projection space:

(12) $$\gamma =\frac{-ln(t)}{\sigma_{o}^{2}},$$

which is a constant for a given sample, with units of ${mrad}^{-2}$ , or the inverse of dark-field imaging units. This equation effectively assumes a macroscopically homogeneous sample. We note that this does not imply a homogeneous microstructure, i.e., one without density variations, which would result in a sample with weak scattering. Hence we clarify this as an assumption of macroscopic homogeneity, with the attenuation and dark field being related to one another across the macroscale, but density variations or heterogeneity allowed on the microscale to scatter the radiation. The simplest examples where this is valid are single-material samples [29].

This is inspired by Paganin retrieval [24,51], where the real and imaginary parts of the refractive index are related, which effectively links the phase-contrast and attenuation signals. On first glance this would suggest redundancy between the two contrast channels; however, the much higher signal-to-noise ratio associated with phase-contrast means Paganin retrieval is widespread for synchrotron-based studies in plant science [52], material science [53], battery research [54], and tissue imaging [55]. This assumption has also been used to develop a single-shot approach to phase-contrast with multi-contrast lab-based imaging systems, including the edge-illumination imaging system [45,56].

The requirement for a constant *γ* is not going to be satisfied by all samples; for example, the complementarity between attenuation and dark-field contrast has been well documented [31,57], and is unlikely to hold for multi-material samples. We primarily introduce this as it allows for simplified retrieval with only a single unknown without any further knowledge or approximation about the scatterers. In this work we show retrievals for samples where this assumption holds well, and samples where this is not completely satisfied, but our approach results in meaningful reconstructions.

The second assumption underlying the proposed approach is that attenuation is relatively low, although this is significantly extended beyond that seen with a pure phase object assumption. This allows for the second-order Taylor expansion in Eq. (12) to be a good approximation. Replacing *t* with 1−a above gives the following expression after the Taylor expansion around −ln(1−a) :

(13) $$-ln(1-a)\approx a+\frac{a^{2}}{2},$$

where *a* is the fraction of the beam attenuated by the sample. Note that through the homogeneous material approximation, this is also related to the dark-field signal. Substituting this into Eq. (12) and rearranging gives the following:

(14) $$\sigma_{o}^{2}=\frac{1}{\gamma }\left(a+\frac{a^{2}}{2}\right).$$

Substituting this and t=1−a into Eq. (7) gives the following polynomial:

(15) $$0=\left(\gamma \Omega -\frac{1}{2}\right)\;a^{2}-\left(2\gamma \Omega +1\right)a+\left(\gamma \Omega -\gamma \sigma_{r}^{2}\right),$$

which is a quadratic whose roots can be found as

(16) $$a=\frac{\left(2\gamma \Omega +1\right)\pm \sqrt{{\left(-2\gamma \Omega -1\right)}^{2}-4\left(\gamma \Omega -\frac{1}{2}\right)\left(\gamma \Omega -\gamma \sigma_{r}^{2}\right)}}{2\gamma \Omega -1},$$

where it was the solution with the negative square root, which was found to give the solution that best matched the expected dark-field signal due to the performance in the limit of $\gamma =0\;{mrad}^{-2}$ . Both Eqs. (14) and (16) can be applied pixel-wise and used to solve for a dark-field image. Estimates for *γ* for each sample can be acquired from conventional retrievals using fully sampled datasets at a first angular projection, which provides averages of *t* and $\sigma_{o}^{2}$ . It will approximate the true dark-field signal if (i) transmission is high, (ii) a homogeneous object can be assumed, and (iii) *γ* is correctly estimated. The first point holds up to where the Taylor expansion approximation around Eq. (12) starts to fail, but potentially more terms could be included for the more attenuating samples. The second and third requirements are more difficult to achieve with non-trivial samples. In practice, the single-shot retrieval is unlikely to yield a quantitative dark-field retrieval across the full image, but a qualitative tomographic reconstruction from a linear single-shot signal showing mixed attenuation and dark-field contrast is achievable with this approach. This is the origin of calling this single-shot retrieval a hybrid of the dark-field and attenuation signals when we make use of this linearization method.

We tested this approach on an attenuating wedge of uniform material, chosen as paper, with profiles shown in Fig. 3. The dimensions of the wedge are roughly 14.8×10mm along the *x* and *z* axes, respectively. At the energy employed here, the refractive index terms are $\delta =5.52\times 10^{-7}$ and $\beta =3.59\times 10^{-10}$ [47]. This was again scanned with 16 dithering steps and 1.2 s exposure per frame, with nine illumination curves taken centered around the peak for conventional dark-field retrieval, with the peak measurement used for single-shot retrieval. When assuming a pure phase object, the signal is non-linear, and additionally orders of magnitude higher intensity than the conventionally retrieved dark-field signal. This can be corrected for by using the linearization approach with Eqs. (14) and (16), which was successful in linearizing the signal and approximating the conventionally retrieved dark-field signal. The paper showed roughly 60% attenuation at its thick edge, and the successful linearization shows that this approach can be used to extend the working region of single-shot retrieval to much more strongly attenuating samples.

## 3. RESULTS AND DISCUSSION

We demonstrate the application of single-shot hybrid contrast to tomography on examples of biological tissues: a rat heart and vascularized endocrine pancreatic tissue. The heart was obtained from the UCL Biological Services Unit, from rats euthanized for organ harvesting via Schedule 1 methods, and critically point dried as per Savvidis *et al.* [58]. The vascularized endocrine pancreas tissue constructs were generated as per the protocol described by Citro *et al*. [59,60], with pancreatic islets embedded in the alveoli of the decellularized lung tissue. The critically point dried tissue was chosen, as dried tissue gives much stronger dark-field contrast compared to that of wet tissue.

Datasets were acquired with eight dithering steps, and 1200 angular views through 360^∘^. A separate heart dataset was acquired with full illumination curve sampling for a comparison with the conventional retrieval approach with seven illumination curve sampling points at 1.2 s per exposure. To match scan time, the heart scan for single-shot retrieval was acquired with 8.4 s per exposure with a single illumination curve sampling point. The pancreatic construct was taken with only 1.2 s per exposure for fast acquisition. The hybrid contrast projections were retrieved from this data using Eq. (14) and reconstructed with the standard filtered back projection algorithm.

**Fig. 4.**
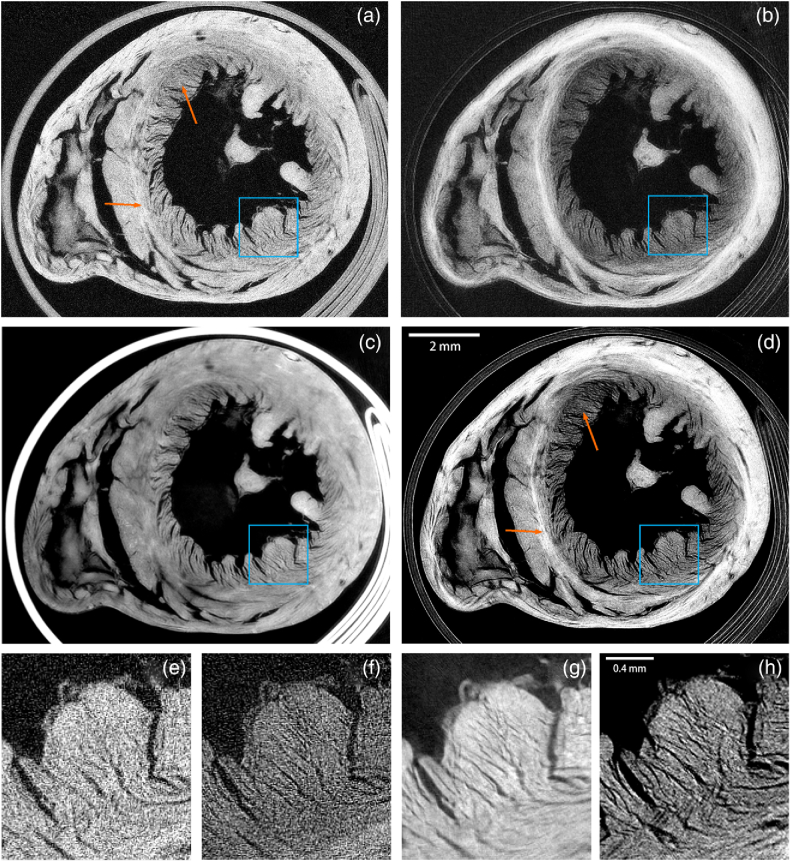
Tomographic slices of dried rat heart sample shown with (a)+(e) attenuation contrast, (b)+(f) dark-field contrast, (c)+(g) phase-contrast, and (d)+(h) hybrid contrast with Eq. (14) and $\gamma =187\;{mrad}^{-2}$ . All slices from datasets with matched total exposure time, with attenuation, phase, and dark field from a fully sampled dataset. Magnified sections in the blue ROI are shown below for the corresponding slices in (e)–(h). The hybrid contrast retains area contrast in the wall (orange arrows) from the dark-field slice, not present in the attenuation slice. Additionally, the inserts show much more detail with hybrid contrast than attenuation contrast.

All samples were successfully reconstructed with no obvious cupping artifacts that would be seen from poor signal linearity (see Supplement 1 Fig. S1 for the effect of carrying out a reconstruction with an incorrect *γ* parameter). Figure 4 shows an axial slice through the middle of the rat heart sample. Dark-field contrast leads to intensity variations in the heart wall due to the different orientations of muscle fiber [31]. The hybrid contrast tomography image appears sharper and richer in detail. A Fourier ring correlation analysis with a three-sigma threshold criterion resulted in an FIRE value (see Ref. [61]) of 52 µm for the hybrid contrast slice, 55 µm for the dark-field slice, 60 µm for the attenuation slice, and 28 µm for the phase-contrast slice. This is partly due to the enhanced contrast at tissue-air interfaces associated with dark-field imaging, but also the improved statistics of single-shot retrieval over conventional retrieval that comes with imaging at the peaks rather than sampling across the full illumination curve, as counts are lower towards the tails. Another possible contribution to the hybrid contrast slice is edge enhancement from the differential phase (refraction) signal that occurs particularly strongly at edges and material boundaries, which our model does not capture as we have low sensitivity to this signal. We additionally show the phase-contrast slice calculated by integrating the refraction signal, with this slice showing a similar level of detail to the hybrid contrast. However, the correlation between these channels can be misleading, as it is the differential phase that might contribute to the hybrid contrast slice, which is a signal that cannot be reconstructed into a tomographic slice.

**Fig. 5.**
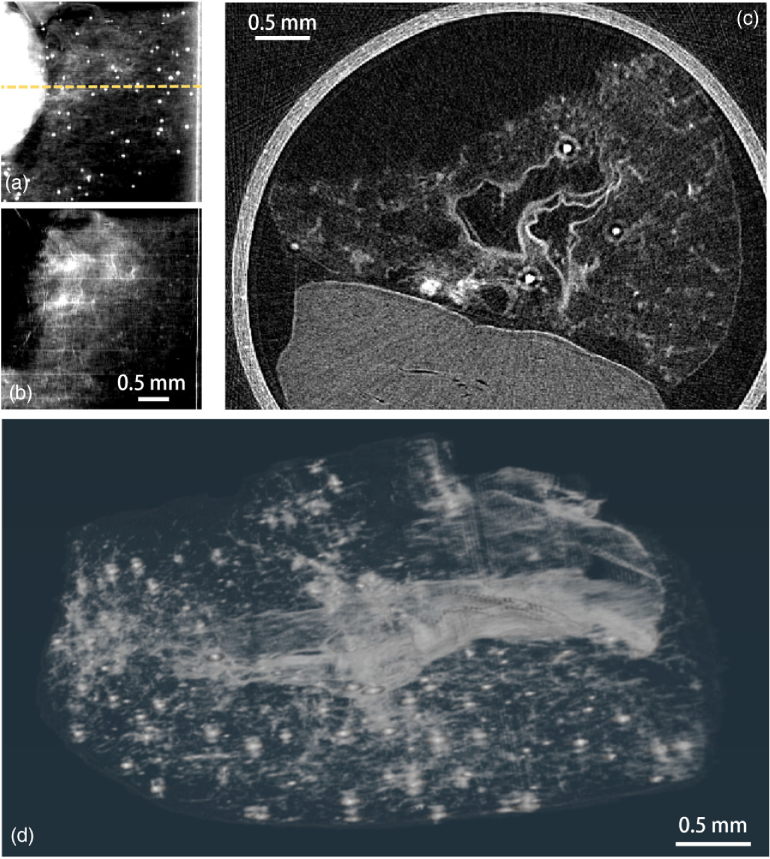
Vascularized endocrine pancreatic tissue sample composed of pancreatic islets embedded in a lung tissue construct. Projection taken with full illumination curve sampling retrieved with (a) attenuation contrast shows the spatial distribution of the islet cells and (b) dark-field contrast shows the lung tissue structure. In (c), a tomography slice at yellow dashed line from a hybrid contrast retrieval with Eq. (14) and $\gamma =138\;{mrad}^{-2}$ . This combines information from both channels and can be visualized as (d) a full volume of joint contrast tomographic scan.

The pancreatic construct is shown in Fig. 5 where a bright layer on the inner surface is due to a strong scattering originating there. The bright spots are round-shaped pancreatic islets, and from a full illumination curve planar image, were found to show a high-attenuation signal. Simultaneous sensitivity to both contrast channels enables concurrent visualization of the islet and the subtle changes in porosity throughout the decellularized scaffold.

All these reconstructions rely on the assumption of insensitivity to the refraction signal when imaging at the peaks of the illumination curve, with the magnitude of the lateral shift in the illumination curve being much smaller than the broadening. From the wedges (Figs. 2 and 3), the magnitude of the refraction signal is much smaller than the square root of the width of the reference illumination curve, i.e., $∥\Delta {\bar{x}}_{ref}∥\ll \sigma_{r}$ , with the latter term being measured at around 8–9 µm depending on the scan. As a quantitative comparison, a strong refraction signal is present at the tissue-air interface for the heart sample of around 2 µm (refraction angle of 12 µrad). Using Eqs. (3) and (4) with typical values of A = 200 and $\sigma_{r}^{2}=81\;\;\text{µ}m^{2}$ , this leads to a drop in peak illumination curve intensity of around 2.5%. The same approach can be used to calculate the same signal and would be found from a purely scattering sample at around $\sigma_{o}^{2}=4\;\;\text{µ}m^{2}(146\;\;\text{µ}{rad}^{2})$ , whereas the measured dark-field signal at this region of the sample is around an order of magnitude higher. There remains, however, a degree of uncertainty in this assessment because the magnitude of refraction signals is ultimately sample-dependent, and thus difficult to generalize beyond the observed cases. Overall, we believe the validity of our assumption and the general presence of dark-field signal at material boundaries means that we are safe to ignore refraction effects.

One area that may affect this single-shot approach is potential contributions of beam hardening. As with x-ray attenuation, dark-field scattering falls with increasing x-ray energy, and hence beam hardening will lead to cupping artifacts with the dark-field reconstruction. The effects of beam hardening on dark-field retrieval have been presented with systems based on interferometric methods [62–64], but not yet with edge-illumination. Furthermore, when combining both contrast channels, beam hardening is likely to lead to a similar problem as with conventional x-ray imaging. The results shown here are with relatively weakly attenuating materials (excluding the paper sample), and hence we do not see clear beam hardening issues in our reconstructions.

From our examples with the wedges and biological tissue, we propose two cases where our approach could be beneficial: (a) where our assumptions hold with *γ* consistent across the whole sample, and secondly (b) where *γ* varies throughout the sample. See Supplement 1 Fig. S2 for an assessment of how *γ* varies within the paper and heart samples. For either case, our approach could result in improved signal-to-noise ratio or contrast when compared to attenuation images, and the benefit of expedited scanning when compared to conventional dark-field acquisition.

In the first case, the attenuation and dark-field signals are linked with a constant ratio between them, resulting in a constant value of *γ*. These are the so-called macroscopic-homogeneous samples. The most basic example of this is the uniform wedge phantoms, where a single material is present and the density and microstructure remain consistent. In theory this could be extended to any sample where the attenuation and dark-field signals are linked, which could be single material samples with varying density of scatterers, or potentially multi-material if each material had the same *γ*. The benefit of using our hybrid contrast for these samples would be if the attenuation signal was poor, such as with the polystyrene phantom. Here, in the projections, the signal-to-noise ratio measured at the thickest part of the wedge rises from 7.0 to 10.8 from the attenuation and hybrid contrast slices, respectively.

The second is for more complex samples where dark-field and attenuation give differing, but somewhat proportional, contrast. This is the case for the heart and pancreas samples, where *γ* varies across the sample. Our retrieval approach uses only a single value across the whole image, and the result is a fusion of the two contrast channels into a hybrid contrast, with information present from both signals. This will be non-quantitative, corresponding to neither dark-field nor attenuation gray values; however the reconstruction can still provide a valid three-dimensional representation of morphology within the sample.

In practice, many complex samples are likely to hold a more complex relationship between the two contrast channels. Although both dark-field scattering and attenuation depend on chemical composite and density, scattering additionally depends on feature size and even orientation [43,65–68]. One example case where our approach is unlikely to hold is that of a material with changing porosity, where optically sparse pores in a uniform media will present reduced attenuation but increased scattering. Different materials are in general expected to have different *γ* parameters; thus multi-material samples are unlikely to satisfy this approach. In future work, our approach could be adapted specifically to samples with multiple *γ* parameters. This is analogous to the extension of Paganin retrieval to multi-material samples [69,70]. These typically require prior knowledge or significant user input and as such, we believe our simple linear relationship provides a suitable retrieval approach for many non-trivial samples.

The tomographic reconstructions we present exhibit high signal-to-noise ratios, showcasing discernible features in both contrast channels. However, attributing intensity to either attenuation or dark field becomes challenging without prior knowledge of sample composition or separate datasets with conventionally retrieved images. While our approach sacrifices the ability to isolate these two contrast channels, the combination of both signals into a single reconstructed volume presents the substantial advantage of synthesizing the data into a single-parameter representation for user inspection. This is opposed to most image fusion methods [71–74], which necessitate full sampling of modulation patterns, retrieval of separate contrast channels, and subsequent synthesis into a unified quantity. Our approach conducts fusion at the retrieval stage and is based on the physical properties of the three-dimensional distribution of materials or tissues under investigation.

Furthermore, our approach offers a notable advantage in that it is maximally efficient with respect to the dose delivered to the sample. This arises by enforcing the data collection at the top of the illumination curve where no primary beam is lost at the analyzer stage, and this exists uniformly across the entire field of view. This optimal data acquisition minimizes any loss of intensity after interaction with the sample, attributing intensity loss solely to contrast rather than to the modulation imposed by the analyzer. To illustrate, our approach is 100% efficient, surpassing alternatives such as a three-point retrieval method [15], which achieves 67% efficiency or lower, and uniform sampling along the illumination curve usually working at around 50% efficiency [30]. We think this is an important point towards the development of X-ray phase-contrast techniques compatible with pre-clinical and clinical imaging.

The versatility of this approach to visualize three different biological samples shows its efficacy to the volumetric investigation of tissue samples. The compact nature of the edge-illumination system, coupled with its utilization of incoherent radiation, makes it particularly well-suited for integration into a cabinet-based design. We believe employing the edge-illumination system with the proposed technique is well-suited for high-throughput analysis of tissue samples in laboratory or clinical settings. The feasibility of such use has been demonstrated before with breast tissue samples using a single-shot phase-contrast approach [32], with our proposed method extending the applicability to imaging scattering tissue.

## 4. CONCLUSIONS

We presented a model and method to obtain X-ray dark-field contrast with a single exposure, without the need for a high spatial or temporal coherence or a high-resolution detector. We derived an equation to retrieve the dark-field signal, which can be found in a single X-ray intensity measurement on pure-phase objects. This was then extended to tomography with more attenuating samples, demonstrating its effectiveness up to approximately 60% attenuation. Our approach for attenuating samples relies on a macroscopically homogeneous material approximation that enables linking the attenuation and dark-field signals within a sample and thus solving for two unknowns with only one measurement. In practice this results in an image with mixed dark-field and attenuation contrast (hybrid contrast), with features from both channels being retained in a single-joint volume. Experimental validation of our approach was conducted on a laboratory-based edge-illumination imaging system, where the use of two absorbing masks allows sensitivity to dark-field contrast in a single shot through an intensity drop arising from scattered X-rays filtered out by the detector mask. A significant advantage of our approach is the reduction of the problem dimensionality, particularly valuable for data-intensive applications like tomographic imaging. We demonstrated the efficacy of our method through using this hybrid contrast for tomography on two biological tissue samples, where the power of dark-field contrast to highlight sub-resolution features was retained with fast and dose-efficient data acquisition. We believe this is a powerful method for obtaining high-quality images while, at the same time, removing the necessity of acquiring multi-dimensional datasets for the extraction of dark-field contrast.

## Supplemental information

Supplement 1 supplemental document https://doi.org/10.6084/m9.figshare.27606141

## Data Availability

Data underlying the results presented in this paper are not publicly available at this time but may be obtained from the authors upon reasonable request.

## Supplemental document

See Supplement 1 for supporting content.
