# Supplementary material for: Hybrid dark-field and attenuation contrast retrieval for laboratory-based X-ray tomography
Source: Optica. 2024 Nov 25;11(12):1603–13. doi: 10.1364/OPTICA.525760 (PMC11674740; doi:10.1364/OPTICA.525760)
Supplement: Supplementary file 1 [file optica-11-12-1603-s001.pdf]

Supplemental Document

OPTICA

Hybrid dark-field and attenuation contrast retrieval for laboratory-based X-ray tomography: supplement

ADAM DOHERTY,[1,2,*] IAN BUCHANAN,[1] ORIOL ROCHE I MORGÓ,[1,2] ALBERTO ASTOLFO,[1] SAVVAS SAVVIDIS,[1] MATTIA F. M. GERLI,[3,4] ANTONIO CITRO,[5] ALESSANDRO OLIVO,[1] AND MARCO ENDRIZZI[1,2]

[1]*Department of Medical Physics and Biomedical Engineering, University College London, London, WC1E 6BT, UK*
[2]*X-ray Microscopy and Tomography Lab, The Francis Crick Institute, 1 Midland Road, London, NW1 1AT, UK*
[3]*UCL Division of Surgery and Interventional Science, Royal Free Hospital, NW3 2PF, London, UK*
[4]*Stem Cell and Regenerative Medicine Section, Great Ormond Street Institute of Child Health, University College London, London, WC1N 1EH, UK*
[5]*San Raffaele Diabetes Research Institute, IRCCS San Raffaele Scientific Institute, Milan, Italy*
[*]*adam.doherty@ucl.ac.uk*

This supplement published with Optica Publishing Group on 25 November 2024 by The Authors under the terms of the Creative Commons Attribution 4.0 License in the format provided by the authors and unedited. Further distribution of this work must maintain attribution to the author(s) and the published article's title, journal citation, and DOI.

Supplement DOI: https://doi.org/10.6084/m9.figshare.27606141

Parent Article DOI: https://doi.org/10.1364/OPTICA.525760

Supplemental Material: Hybrid dark-field and attenuation contrast retrieval for laboratory-based x-ray tomography

ADAM DOHERTY[1,2,*], IAN BUCHANAN[1], ORIOL ROCHE I MORGÓ[1,2], ALBERTO ASTOLFO[1], SAVVAS SAVVIDIS[1], MATTIA F. M. GERLI[3,4], ANTONIO CITRO[5], ALESSANDRO OLIVO[1] AND MARCO ENDRIZZI[1,2]

[1] *Department of Medical Physics and Biomedical Engineering, University College London, London, WC1E 6BT, UK*
[2] *X-ray Microscopy and Tomography Lab, The Francis Crick Institute, 1 Midland Road, London, NW1 1AT, UK*
[3] *UCL Division of Surgery and Interventional Science, Royal Free Hospital, NW3 2PF, London, UK*
[4] *Stem Cell and Regenerative Medicine Section, Great Ormond Street Institute of Child Health, University College London, London, WC1N 1EH, UK*
[5] *San Raffaele Diabetes Research Institute, IRCCS San Raffaele Scientific Institute, Milan, Italy*
[*] *adam.doherty@ucl.ac.uk*

S1. Introduction

Here we present supplemental material for "Hybrid dark-field and attenuation contrast retrieval for laboratory-based tomography". We focus on the choice of γ for various samples. Section S2 investigates the effects of choosing an incorrect gamma, where cupping artefacts are seen if γ is significantly wrong, but these remain mild for the relatively weakly attenuating heart sample. Section S3 discusses how γ varies within the paper and heart samples. For the single-material paper, the bulk-homogeneous approximation holds and γ remains consistent. For the heart sample, our approximation breaks down, but due to our relative robustness to differing γ as seen in Section S2, we can still use a single value for γ to produce a non-quantitative reconstruction without artefacts.

S2. Sensitivity to γ

For attenuating samples, the single-shot signal must be linearised for tomographic reconstruction. The procedure for this involves choosing a material parameter, γ, that relates the expected attenuation and dark-field signals. We propose that for tomographic acquisition, full illumination curve sampling and retrieval should be carried out to measure γ at a single projection. However, γ is likely to somewhat vary within the sample and thus it is important to understand the effects of incorrect choice of γ.

We tested the linearity of the signal from the paper wedge phantoms. This was done with simulated Gaussian functions to remove noise and other potential errors, here we concentrate on the robustness of the model our approach is based on. These Gaussian functions were defined to have an area reduction (i.e. attenuation signal) from 0 to 1.1, and a broadening (dark-field signal) of 0 to 55 μm^2 (0 to 2000 μrad^2), which were the range seen with the real phantom data. The retrieved signals were calculated for $\gamma = 0$ mrad^{-2} (which is the assumption of a pure phase object), $\gamma = 138$ mrad^{-2} (underestimation), $\gamma = 551$ mrad^{-2} (correct value), and $\gamma = 5510$ mrad^{-2} (overestimation). Profiles for the retrieved single-shot signal are shown in Fig. S1, with the intensity rescaled due to the drastically different intensity that would be otherwise measured.

The profiles in Fig. S1 show that the profile with an underestimated γ is non-linear and thus would cause significant cupping artefacts if used for tomographic reconstruction. This is expected, as this is the same as assuming $t = 1$ with the non-linearised signal in the main manuscript. Alternatively, an overestimated γ causes a sub-linear signal with lower intensity than the true

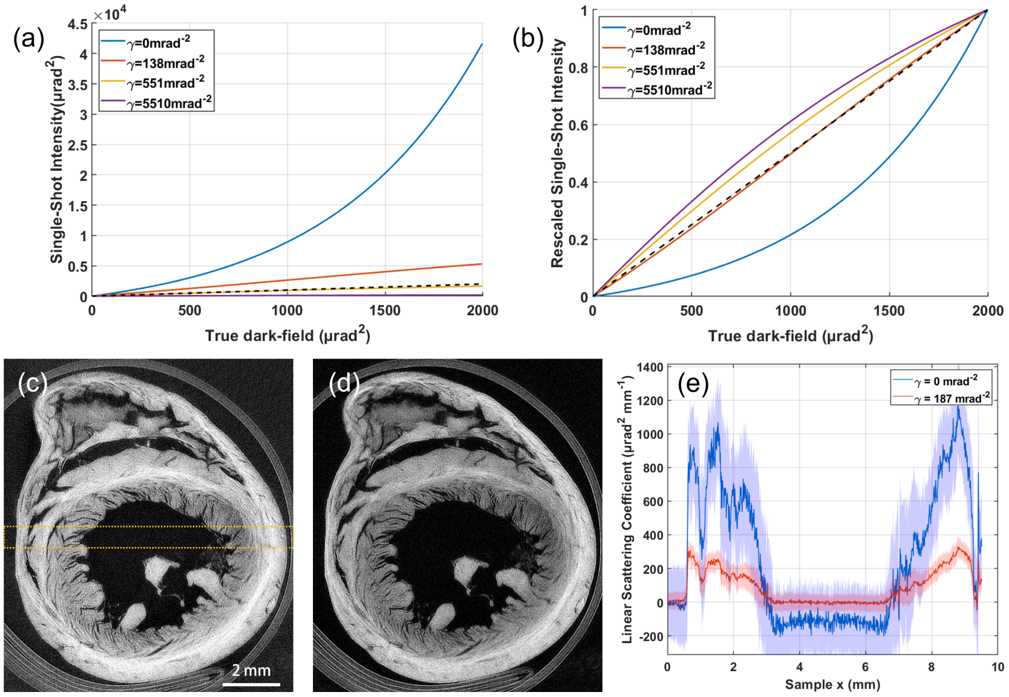

Fig. S1. Profiles of retrieved single-shot signal from the simulated Gaussian functions to match the signal from the paper wedge phantom (a) raw retrieved signal to show accuracy and (b) rescaled retrieved signal to show linearity. The yellow line is the true value of γ and the black dotted line is a line of equality. Tomographic slices of the heart sample with signal retrieved with (c) $\gamma = 0mrad^{-2}$ and (d) $\gamma = 187mrad^{-2}$ (true value). Profiles in (e) around the orange ROI show that incorrect choice of γ leads to incorrect intensity values and cupping artefacts - indicated by the negative values in the ventricular space.

dark-field signal, and again cupping artefacts would be seen. The true γ value brings about a good agreement with the true dark-field signal, however, a slightly non-linear behaviour is seen, likely due to the breakdown of a moderately attenuating sample. For this sample where attenuation is high, a slight underestimation of γ can give a more linear signal, but the intensity values here are significantly above those of the true dark-field signal

We then extended this to tomography to show these artefacts. The single-shot signal from the heart sample was retrieved with $\gamma = 0$ mrad^{-2} and $\gamma = 187$ mrad^{-2}. These show that when gamma is incorrectly estimated, the empty ventricular space appears darker than the background, caused by the cupping artefacts. Note that there should not be any resolution changes with varying γ that are associated with Paganin phase retrieval, as we do not work in Fourier space.

These results show that for moderately attenuating samples such as the heart, an incorrect choice for γ results in cupping artefacts, although these are still mild. A correct choice brings about a signal without artefacts, with an intensity which well matches that retrieved using conventional dark-field retrieval.

S3. Sample uniformity

As shown in the previous section, an incorrect choice of γ can lead to artefacts in the reconstruction. As only a single value of γ is used for retrieval across all pixels in all projections,

quantitative dark-field retrieval is not possible with macroscopically-inhomogeneous objects where γ differs within the sample. Here, we assess the variability of this parameter within the macroscopically-homogeneous paper sample and the macroscopically-inhomogeneous heart sample. The polystyrene wedge is not shown due to the low signal leading to high levels of noise.

For this assessment, we retrieve the true attenuation and dark-field signals from the full illumination curve sampled scans mentioned in the main manuscript. Afterwards, we divide the two images to create a map of γ. The attenuation images had the sample segmented before calculating γ to avoid instabilities in the background. Note that the masks are strengthen using horizontal bridges that result in stripes in the image. These are typically faint but seem to be magnified when showing images of γ.

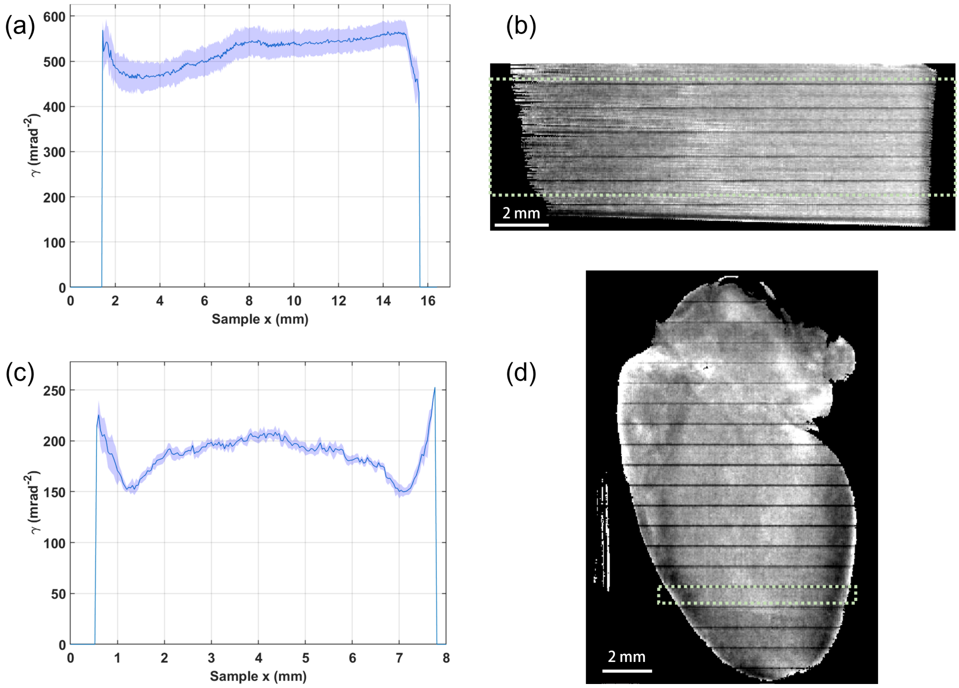

Fig. S2. The variability of γ within the (a-b) paper and (c-d) rat heart samples, with profiles plotted around the green ROIs. The paper is a single material and hence a macroscopically homogeneous sample. The heart is not such a sample due to the complementary signal from the two channels.

For the paper sample, which consists of a single material, γ remains generally consistent throughout the sample. There is a discrepancy of roughly 10% between the two ends of the wedge - the origin of which is not known, but could simply be a slightly imperfect phantom. Even so, this level of variation is within the order of magnitude variation required for significantly non-linear signal shown in Section S2. Furthermore, the spatial variation of this signal is largest in the thinner parts of the sample - which not only suggests this could also originate from noise, but the linearisation approach is less important in the weakly attenuation areas of the sample. The average of this profile of 551 mrad^{-2} was used for linearisation of the single-shot signal.

For the heart sample, a larger variation of roughly 25% is present due to the significant difference in contrast between the attenuation and dark-field projections. This is most pronounced towards the edge of the sample, where the heart wall is thickest, and we see the fibres bringing a higher dark-field signal (and hence lower γ). Once again this variation falls within the variation that is unlikely to significantly affect signal linearity. A single choice of γ = 187 mrad^{-2} results

in a non-quantitative retrieval of the dark-field signal, which becomes mixed with the attenuation and creates the hybrid contrast for tomography reconstruction.
